# Dataset on viscosity and starch polymer properties to predict texture through modeling

**DOI:** 10.1016/j.dib.2021.107038

**Published:** 2021-04-03

**Authors:** Reuben James Q. Buenafe, Vasudev Kumanduri, Nese Sreenivasulu

**Affiliations:** a Grain Quality and Nutrition Center, International Rice Research Institute, Los Baños, Laguna 4031 Philippines; b School of Chemical, Biological and Materials Engineering and Sciences, Mapua University, Muralla St., Intramuros, Manila 1002 Philippines; c Piatrika Biosystems, Cambridge, United Kingdom

**Keywords:** Cooking and eating quality, Random forest model, Indica, Rice properties

## Abstract

Accurate classification tool for screening varieties with superior eating and cooking quality based on its pasting and starch structure properties is in demand to satisfy both consumers’ and farmers’ need. Here we showed the data related to the article entitled “Deploying viscosity and starch polymer properties to predict cooking and eating quality models: a novel breeding tool to predict texture” [1] which provides solution to this problem. The paper compiles all the pasting, starch structure, sensory and routine quality data of the rice sample used in the article into graphical form. It also shows how the data were processed and obtained.

## Specifications Table

**Table:** 

Subject	Data Science
Specific subject area	Multivariate Analysis
Type of data	Table, Graph, Chart, Figure
How data were acquired	Amylose content (AC) of rice was obtained using San++ Segmented Flow Analyser (SFA) system (Scalar analytical B.V., AA Breda, Netherlands) through colorimetric analysis. Gelatinization temperature (GT) was determined using Differential Scanning Calorimetry Q100 instrument (TA Instrument, New Castle, DE, USA). Gel consistency (GC) was obtained through measuring the length of cold paste of rice flour. The pasting properties (PsT, PkT, PV, TV, FV, BD, SB and LO were captured using Rapid visco-analyzer (RVA, (Model 4-D, Newport Scientific, Warriewood, Australia) while the starch structure composition (AM1, AM2, MCAP, SCAP1, SCAP2,and SCAP3) were obtained by debranching with isoamylase (Pseudomonas, Megazyme, Wicklow, Ireland) and analyzing using size exclusion chromatography (SEC) equipped with Ultrahydrogel 250 column (Waters, Alliance 2695, Waters, Millford, USA). Sensory textural data were obtained through the scores of trained panelists. All the processed data were developed using R software (Version 3.3.2, released 2016).
Data format	Analyzed
Parameters for data collection	Amylose content was measured at 620 nm, while the gelatinization temperature was measured at the range of 25 to 120 °C at a rate of 10 °C per minute. RVA properties were measured from 50 to 90 to 50 °C. Before subjecting to SEC, the samples were debranched at 50 °C for 2 h.
Description of data collection	The rice was used as received from harvest. It undergone milling and turned into powdered form. The routine quality parameters (AC, GT, and GC) were collected using established protocols at IRRI. The RVA properties were analyzed using the AACC method 61–02. SEC data were also collected for the starch structure composition of the rice samples.
Data source	International Rice Research Institute, Los Baños, Laguna, Philippines
Data accessibility	Buenafe, Reuben James; Sreenivasulu, Nese (2021), “Supplementary Materials for Dataset on viscosity and starch polymer properties to predict texture through modeling”, Mendeley Data, V2, https://doi.org/10.17632/xkzx3xhj2y.2
Related research article	R.J.Q. Buenafe, V. Kamanduri, N. Sreenivasulu, Deploying viscosity and starch polymer properties to predict cooking and eating quality models: a novel breeding tool to predict texture, Carbohydrates Polymer. (2021) 117,766. https://doi.org/10.1016/j.carbpol.2021.117766[1]

## Value of the Data

• The data are useful as a guide on how to utilize the physico-chemical properties of cooked rice as classification predictors in relation to consumer perception.
• This could benefit breeding programs in selective breeding targeted towards both the consumers’ and farmers’ preferences since the data provides various way of rice classification through the use of different physico-chemical properties of cooked rice.
• The data presented in this paper could be used as template in classifying rice based on other perceived parameters such as taste or flavor, aroma, and appearance.

## 1 Data Description

**Fig. 1:**
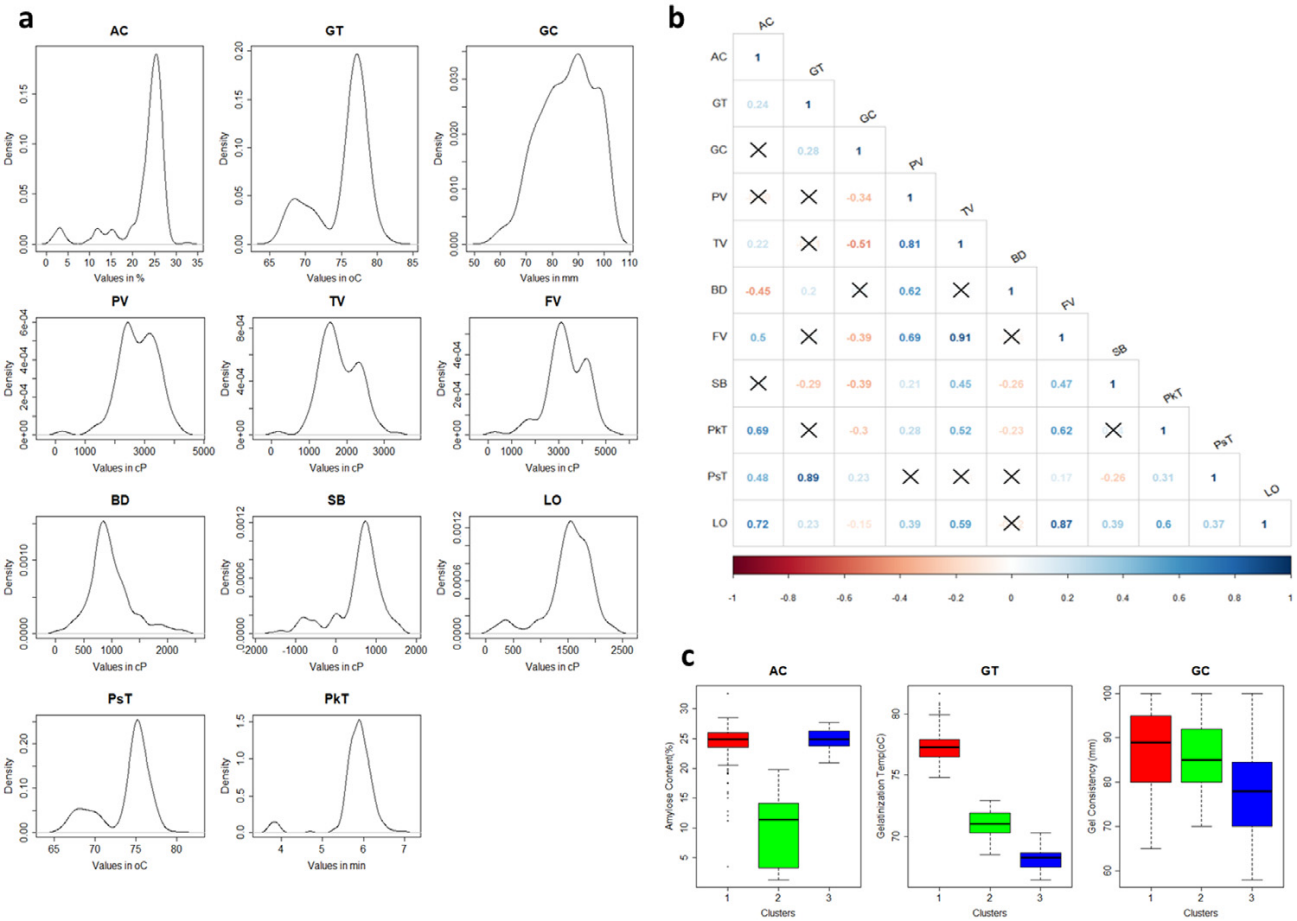
Phenotypic distribution and correlation of selected indica lines (*n* = 301) according to the eating and cooking quality. (a) Kernel density distribution based on routine grain quality parameters and rapid-visco analyzer (RVA) pasting properties. (b) Correlation relationships of cooking and eating quality parameters. The boxes with X marks were considered insignificant at p-value = 0.05 while the red and blue colors show positive and negative correlations, respectively. (c) Boxplots comparing the three clusters based on routine grain quality parameters. Variable names were as follows: amylose content (AC), gelatinization temperature (GT), gel consistency (GC), peak viscosity (PV), trough viscosity (TV), breakdown viscosity (BD), final viscosity (FV), setback viscosity (SB), peak time (PkT), pasting temperature (PsT) and lift-off viscosity (LO).

**Fig. 2:**
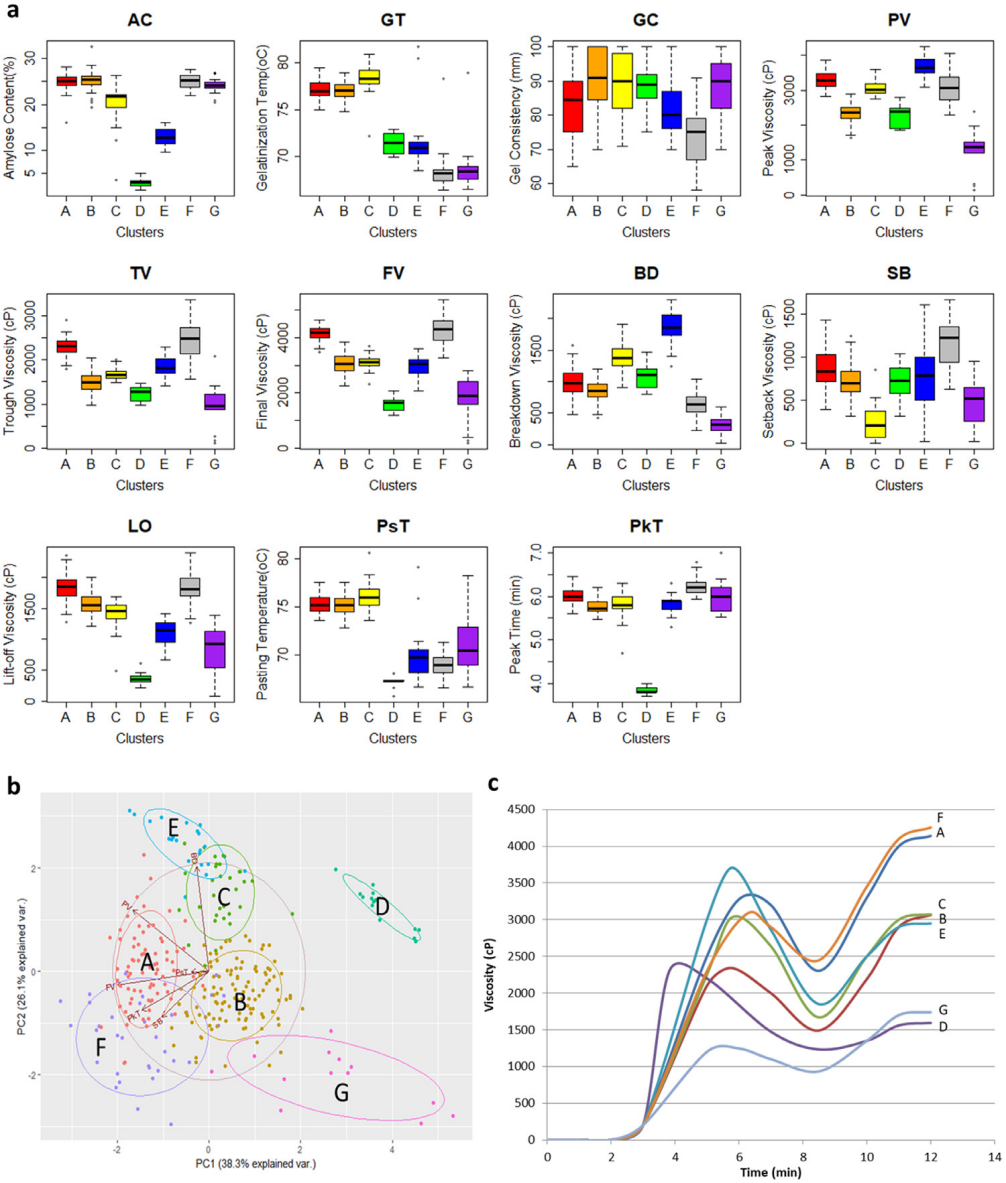
Phenotypic variance of selected indica lines (*n* = 301) within the seven clusters. (a) Boxplots comparing the routine grain quality parameters of seven clusters created based on selected RVA parameters. (b) Principal component analysis biplot for PC1 (38.3% explained variability) and PC2 (26.1% explained variability). (c) RVA viscosity profile of the seven clusters.

**Fig. 3:**
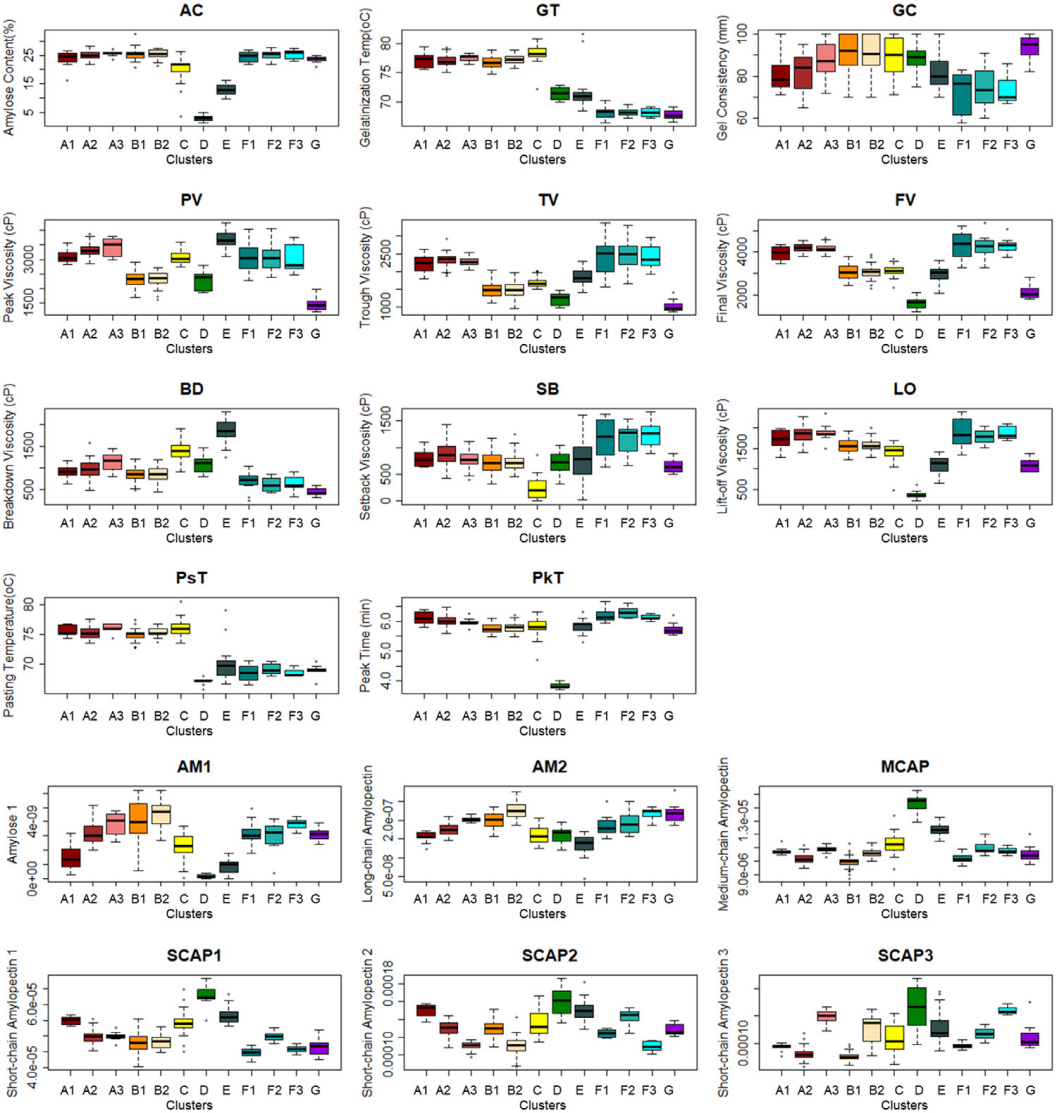
Phenotypic variance of selected indica lines (*n* = 301) within the twelve clusters. Variable names were as follows: amylose content (AC), gelatinization temperature (GT), gel consistency (GC), peak viscosity (PV), trough viscosity (TV), breakdown viscosity (BD), final viscosity (FV), setback viscosity (SB), peak time (PkT), pasting temperature (PsT) and lift-off viscosity (LO), AM1 (Amylose 1), AM2 (Long-chain Amylopectin), MCAP (Medium-chain Amylopectin), SCAP1 (Short-chain amylopectin, 36>DP>21), SCAP2(Short-chain amylopectin, 20>DP>13), SCAP3 (Short-chain amylopectin, 12>DP>6).

Fig. 1 shows the phenotypic distribution of the rice diversity Indica panel (*n* = 301) based on 11 cooking and eating quality parameters. This includes both routine quality [amylose content (AC), gel consistency (GC), and gelatinization temperature (GT)] and rapid-visco analyzer (RVA) properties [peak viscosity (PV), trough viscosity (TV), final viscosity (FV), pasting temperature (PsT), peak time (PkT), breakdown viscosity (BD) and lift-off viscosity (LO)]. Fig. 1 also shows the relationship of all these parameter through correlation analysis. Moreover, cluster distribution of each line of Indica rice when they were grouped together through agglomerative nesting by Ward's method (AGNES) using the routine quality properties were also presented in Fig. 1. The data for the cooking and eating quality parameters, from which Fig. 1 was derived, were provided in the Supplementary Material 1 [S1]. S1 shows the average values of all the triplicate runs for each analysis. Fig. 2 shows the phenotypic distribution of the Indica lines in seven cooking and eating quality ideotypes. The ideotypes were identified using the RVA properties presented in S1. Fig. 2 also shows the distinctness of each ideotype though Principal Component Analysis (PCA) and its RVA viscosity profile. The data used for the RVA viscosity profile presented in Fig. 2 were provided in the Supplementary Material 2 [S2]. S2 gives the average viscosity profile of the triplicate run of each sample analyzed. Fig. 3 shows the phenotypic distribution of the data in 12 cooking and eating quality classes derived from the reclassification of seven clusters using the starch structure composition values found after size-exclusion chromatography (SEC) analysis. The starch structure compositions used were AM1 (Amylose 1), AM2 (Long-chain Amylopectin), MCAP (Medium-chain Amylopectin), SCAP1 (Short-chain amylopectin, 36>DP>21), SCAP2 (Short-chain amylopectin, 20>DP>13), SCAP3 (Short-chain amylopectin, 12>DP>6) which were all presented in S1. The data for the average of triplicate runs for each sample were presented in Supplementary Material 3 [S3]. The sensory profile scored by the panelist and their corresponding descriptions were summarized in Table 1 while the average scores for each ideotype were presented in Table 2. The average for each lines used for Table 2 were provided in S1while the raw scores provided by each panelist were presented in Supplementary Material 4 [S4].

**Table 1:** Sensory textural attributes considered in the study.

			Vocabulary used to describe	
Sensory Attribute	Abbreviation	Description	Minimum Score	Maximum Score
Initial Starchy Coating	ISC	Amount of paste-like thickness perceived on cooked rice grain before mixing with saliva up to three passes.	Not Starchy	Starchy
Slickness	SLK	Maximum ease of passing tongue over the cooked rice grain surface when saliva starts to mix with the sample.	Not Slick	Slick
Roughness	ROF	Amount of irregularities on the surface of cooked rice grain.	Smooth	Rough
Stickiness to Lips	STL	Degree to which the cooked rice grain adhere to the inner lip.	Not Sticky to Lips	Sticky to Lips
Stickiness Between Grains	SBG	Degree to which the cooked rice grain adhere to each other.	Loose	Compact
Springiness	SPR	Degree cooked rice grain returns to original shape after partial compression.	Stiff	Springy
Cohesiveness	COH	Degree to which the grains deform rather that crumble, crack or break when biting with molars.	Breakable	Resilient
Hardness	HRD	Amount of force required to bite the cooked rice grain with the molars.	Soft	Hard
Cohesiveness of Mass	COM	Maximum degree to which the cooked rice grains hold together in a mass while it is chewed.	Crumbly	Cohesive
Uniformity of Bite	UOB	Evenness of force throughout bites to chew.	Varying Bite	Uniform Bite
Moisture Absorption	MAB	Amount of saliva absorbed by the sample as it is chewed.	Moist	Dry
Residual Loose Particles	RLP	Amount of loose particles left inside the mouth after swallowing.	Low Residue	High Residue
Toothpack	TPK	Amount of cooked rice grain adhering in or on the teeth.	Low TPK	High TPK

**Table 2:** Average scores of sensory panel for each cluster.

	Sensory Scores												
Cluster	ISC	SLK	ROF	STL	SBG	SPR	COH	HRD	COM	UOB	MAB	RLP	TPK
A1	70±21	55±14	48±13	43±6	51±5	47±12	51±10	45±8	59±21	72±23	62±6	64±24	35±11
A2	55±17	67±10	44±8	45±14	51±10	45±10	52±15	49±6	60±16	83±18	64±7	72±11	33±14
A3	32±12	64±8	38±5	53±19	46±15	48±8	45±10	43±9	55±6	75±3	62±7	73±3	27±12
B1	58±21	54±14	49±12	42±15	56±17	48±6	48±9	51±9	64±16	76±11	66±8	73±15	45±14
B2	58±22	58±14	52±9	55±21	54±10	52±8	56±10	51±4	63±10	78±8	69±9	71±9	49±13
C	68±27	66±17	38±11	84±31	76±23	47±12	61±18	40±11	91±16	100±17	67±13	54±18	50±20
D	118±17	57±25	37±12	136±10	121±16	23±6	69±11	21±5	117±11	129±7	58±15	36±19	61±18
E	102±15	59±14	36±5	113±17	102±16	43±21	71±16	29±8	101±5	116±6	63±14	32±14	62±12
F1	41±23	72±1	40±5	54±9	55±22	52±15	61±14	50±15	63±14	77±20	66±3	80±15	51±15
F2	65±22	67±11	48±10	49±14	62±18	44±5	47±14	52±8	62±17	78±17	66±11	63±11	54±22
F3	25±22	60±9	58±17	43±16	39±18	40±5	43±9	52±7	42±22	65±13	66±8	74±11	49±32
G	43±24	49±8	59±18	41±18	53±17	44±4	38±2	56±3	69±16	75±19	67±10	74±11	55±8

## 2 Experimental Design, Materials and Methods

### 2.1 Rice varieties

A (*n* = 301) set of indica rice accession seed was selected according to their geographic distribution and genetic diversity, planted and grown during the dry season of 2014 at International Rice Research Institute (IRRI) under field conditions. After harvesting the grains and reducing it to 14% moisture content, they were dehulled (Rice sheller THU-35A, satake Corporation, Hiroshima, Japan) and milled (Grainman 60–230–60–2AT, Grain Machinery Mfg. Corp., Miami, USA). Some of the grains were converted to powdered (Cyclone Sample Mill 3010–039, Udy Corporation, Fort Collins, USA) to undergo various biochemical analyses.

### 2.2 Determination of AC, GT, and GC

The AC, GT and GC have been routinely determined to identify the quality of rice grains. Traditionally, AC was determined through standard iodine colorimetric method (ISO 6647–2–2011) [2]. In this method, a sample of rice flour (100 mg) was suspended in 95% ethanol (1.0 mL) then added with 1.0 N NaOH (9.0 mL). The mixture was heated for ten minutes on a 95 °C water bath then cooled to room temperature and diluted with deionized water (100 mL). The sample was reacted with 1.0 N CH_3_COOH (10% w/v) and 2%:0.2% KI-I_2_ solution (30% w/v) and the absorbance were measured at 620 nm in San++ Segmented Flow Analyser (SFA) system (Scalar analytical B.V., AA Breda, The Netherlands). The AC was quantified using a standard calibration curve from standard rice reference (IR65, IR24, IR64 and IR8). These were run in triplicates.

The GT was determined in triplicates using differential scanning calorimetry (DSC Q100 instrument, TA Instrument, New Castle, DE, USA) [3]. Rice flour (4.0 mg) was immersed in Millipore water (8.0 mg) and hermetically sealed and heated from 25 °C to 120 °C at a rate of 10 °C per minute.

GC was determined in triplicates using length measurement of the cold paste. Rice flour (100 mg) was mixed with ethanol (0.2 mL) containing 0.025% thymol blue and 0.2 M KOH (2 mL) and heated for 8 min in a boiling water bath. The mixture was cooled immediately in an ice-water bath and laid down horizontally for one hour [2], [4]. The length was compared to GC standards for hard (IR48), medium (PSBRC9), and soft (IR42).

### 2.3 Pasting properties and starch structure properties

The pasting properties of cooked rice were determined through the AACC method 61–02 [5]. Rice flour (3.0 g) was suspended in reverse-osmosis (RO) purified water (25 g) in a canister and was subjected to RVA (Model 4-D, Newport Scientific, Warriewood, Australia). The temperature setting was ramped from 50 to 95 °C then eventually cooled to 50 °C. ThermoCline for Windows (TCW) version 2.6 was used to collect and record the data to develop the viscosity profile [6]. The readings were done for triplicates of samples.

The starch structure properties were determined using size exclusion chromatography (SEC) with Ultrahydrogel 250 column (Waters, Alliance 2695, Waters, Millford, USA) [7]. Rice flour (50 mg) was gelatinized and debranched with 500 U/mL of isoamylase (Pseudomonas, Megazyme, Wicklow, Ireland) for 2 h at 50 °C while stirring continuously. An aliquot (40 µl) of this solution was then analyzed in the SEC in triplicates.

### 2.4 Sensory evaluation

A set of samples (*n* = 110) from the rice diversity Indica panel was chosen and were cooked as prescribed by Cuevas et al. [8], [9]. Trained set of panelist, selected based on their availability was chosen and were cooked as prescribed by Cuevas et al. [8], [9]. Trained set of panelist, selected based on their availability and previous training, evaluated the texture profile of the samples based on the properties presented in Table 1. The panelist undergone various tests during the training phase which includes difference test, sample and method familiarization and vocabulary adjustments based on their own panelists’ contexts [10]. The rice samples used in training were commercially available milled rice such as Sinandomeng, Jasmine and Long Grain Rice.

### 2.5 Multivariate analyses of cooking and eating quality data

All multivariate analyses were done using R software (Version 3.3.2, released 2016). The distribution and variation of the data set was observed using Kernel density plots. Correlational analysis of all the measured variables (routine quality parameters, RVA data, and starch properties) was also done to see the relationships of each variable from one another. Variables with p-value < 0.05 were considered not significant while values with |r| ≥ 0.70 were considered highly correlated.

Preliminary clustering was done using the routine quality parameters (AC, GC, and GT). The clusterability of the data set was assessed at p-value < 0.05 using Hartigan's dip test for pairwise distances [11], [12], [13], [14]. In this test, the pair-wise distances were used to determine the modality of the data. Clusterability was identified if the pairwise distances of the data sets were found to be multimodal (p-value < 0.05) [11]. AGNES was used to cluster all the data. The values of measured variables were rescaled using min-max normalization. The normalized values were used as inputs for the clustering.

The RVA data were used to reclassify the dataset into a more comprehensive cooking quality ideotypes using the same clustering method. PCA was performed using the RVA data of all the samples to see if there is distinct separation between clusters. The scores were obtained and plotted in a bi-plot to see the distinction between the groups created from the clustering. The loadings of each variable were also obtained to see which variable is correlated to each cluster. The created classes or clusters were concluded as the cooking and eating quality ideotypes for the selected lines. Each ideotype were sub-clustered using starch SEC data via AGNES and the created classes were identified as the subclass of each ideotype.

## Declaration of Competing Interest

The authors declare no known competing financial interests or personal relationships which have or could be perceived to have influenced the work reported in this article.

## Appendix Supplementary materials

Image, application 1

## Footnotes

Supplementary material associated with this article can be found in the online version at doi:10.1016/j.dib.2021.107038.
